# The 5′-end motif of Senecavirus A cDNA clone is genetically modified in 36 different ways for uncovering profiles of virus recovery

**DOI:** 10.3389/fmicb.2022.957849

**Published:** 2022-08-17

**Authors:** Hailan Meng, Qi Wang, Meiling Liu, Ziwei Li, Xiaojing Hao, Di Zhao, Yaqin Dong, Shuang Liu, Feng Zhang, Jin Cui, Bo Ni, Hu Shan, Fuxiao Liu

**Affiliations:** ^1^College of Veterinary Medicine, Qingdao Agricultural University, Qingdao, China; ^2^Department of Animal Medicine, Shandong Vocational Animal Science and Veterinary College, Weifang, China; ^3^Surveillance Laboratory of Livestock Diseases, China Animal Health and Epidemiology Center, Qingdao, China; ^4^Qingdao Workstation of Animal Husbandry, Qingdao, China

**Keywords:** Senecavirus A, 5′ terminus, hairpin-pseudoknot-hairpin structure, VPg-pUpU, self-repairing, virus rescue, *cis*-acting replication element

## Abstract

Senecavirus A (SVA) is an emerging picornavirus. Its genome is one positive-sense, single-stranded RNA. The viral protein (VPg) is covalently linked to the extreme 5′ end of the SVA genome. A complex hairpin-pseudoknot-hairpin (HPH) RNA structure was computationally predicted to form at the 5′ end of the SVA genome. A total of three extra “U” residues (UUU) served as a linker between the HPH structure and the VPg, causing putative UUU–HPH formation at the extreme 5′ end of the SVA genome. It is unclear how the UUU–HPH structure functions. One SVA cDNA clone (N0) was constructed previously in our laboratory. Here, the N0 was genetically tailored for reconstructing a set of 36 modified cDNA clones (N1 to N36) in an attempt to rescue replication-competent SVAs using reverse genetics. The results showed that a total of nine viruses were successfully recovered. Out of them, five were independently rescued from the N1 to N5, reconstructed by deleting the first five nucleotides (TTTGA) one by one from the extreme 5′ end of N0. Interestingly, these five viral progenies reverted to the wild-type or/and wild-type-like genotype, suggesting that SVA with an ability to repair nucleotide defects in its extreme 5′ end. The other four were independently rescued from the N26 to N29, containing different loop-modifying motifs in the first hairpin of the HPH structure. These four loop-modifying motifs were genetically stable after serial passages, implying the wild-type loop motif was not a high-fidelity element in the first hairpin during SVA replication. The other genetically modified sequences were demonstrated to be lethal elements in the HPH structure for SVA recovery, suggesting that the putative HPH formation was a crucial *cis*-acting replication element for SVA propagation.

## Introduction

Senecavirus A (SVA) is an emerging virus, also known as Seneca Valley virus (SVV), which causes vesicular disease in swine (Hales et al., 2008). SVA-infected cases have been found in several countries, including Canada, the United States, Brazil, China, Thailand, and Vietnam. Therefore, this emerging virus has attracted a great deal of attention from the pig industry. SVA is assigned taxonomically to the family *Picornaviridae*, genus *Senecavirus*. A mature virion is an icosahedral-shaped particle without an envelope, ~30 nm in diameter (Strauss et al., 2018). The viral capsid is composed of a densely-packed icosahedral arrangement of 60 protomers, each consisting of VP1, VP2, VP3, and VP4, whereas VP4 is located on the internal side of the capsid. The viral genome is one positive-sense, single-stranded RNA, approximately 7,300 nt in length, coding for a single polyprotein precursor, which will be cleaved further into various polypeptides: L, VP4, VP2, VP3, VP1, 2A, 2B, 2C, 3A, 3B, 3C, and 3D (Liu et al., 2021a).

The polyprotein open reading frame (ORF) is flanked by 5′ and 3′ untranslated regions (UTRs), approximately 670 and 70 nt, respectively. The SVA 5′ UTR harbors an internal ribosome entry site (IRES) that allows for translation initiation in a cap-independent manner. The picornaviral IRES elements are commonly classified into five types (Lozano and Martínez-Salas, 2015). The SVA's IRES is classified into the hepatitis C virus-like type (Willcocks et al., 2011). The SVA 3′ UTR was computationally predicted to harbor either a kissing-loop structure, in which two putative loops interacted with each other to form (Hales et al., 2008), or an H-type-like pseudoknot (Liu et al., 2022). The SVA genome is actually an mRNA, containing a 3′ poly(A) tail, but no 5′-capped structure. Instead, the VPg (or 3B) is covalently linked to the 5′ terminus of the picornaviral genome for initiating viral replication by acting as a protein primer for RNA synthesis (Gavryushina et al., 2011; Paul and Wimmer, 2015).

Such a covalent linkage is thought to occur in two steps. First, the VPg serves as a primer for the production of diuridylylated VPg (VPg-pUpU) in a reaction catalyzed by the viral polymerase. The VPg-pUpU is characterized by the covalent linkage of two uridine monophosphate molecules to the hydroxyl group of the third VPg residue (tyrosine), which is highly conserved among picornaviruses. The *cis*-acting replication element (*cre*), located in different regions of the picornaviral genomes, is a specific template for initiating such a covalent linkage (Lobert et al., 1999; Paul et al., 2000; Yang et al., 2002, 2008). Second, the VPg-pUpU is transferred to 3′ termini of plus- and minus-strand RNAs, and then, serves as a primer for generating a full-length RNA (Paul et al., 1998; Pathak et al., 2008; Sun et al., 2014). The molecular characteristics of VPg-pUpU indicate that two consecutive uridines must be the first two nucleotides at the extreme 5′ end of the wild-type SVA genome.

Picornaviral 5′ UTRs generally contain two major functional domains. Besides the IRES, the other one is also a high-order structure, which is located at the 5′ terminus of the genome and is variable among those of the picornaviruses. A well-studied one is a complex structure at the 5′ terminus of the enteroviral genome, where a small (<100 nt) self-complementary region, referred to as the 5′ cloverleaf, exists. The 5′ cloverleaf formation functions as a platform upon which various host and virus proteins assemble to initiate the synthesis of the anti-genome (Andino et al., 1990, 1993; Leong et al., 1993; Parsley et al., 1997; Barton et al., 2001). Therefore, the 5′ cloverleaf structure can be called the replication platform of the enterovirus (Pascal et al., 2020).

Another typical example is a hairpin-hairpin-hairpin (HHH) formation at the 5′ terminus of the Aichi virus (picornavirus). These three consecutive hairpins were demonstrated to be indispensable for viral replication and critical for viral RNA encapsidation (Sasaki et al., 2001; Nagashima et al., 2003; Sasaki and Taniguchi, 2003). Furthermore, an additional structure, pseudoknot formation, was demonstrated to exist across the HHH element. Pseudoknot is a type of high-order RNA structure, formed upon base-pairing of a single-stranded region of RNA in the loop of a hairpin to a stretch of complementary nucleotides elsewhere in the RNA chain (Brierley et al., 2007). The pseudoknot formation was also proven to be necessary for RNA replication of the Aichi virus (Nagashima et al., 2005).

The above-mentioned cloverleaf or HHH structure can be classified into the *cre* category that is required for negative-strand RNA synthesis. Unfortunately, SVA as an emerging virus shows a less-defined function of the RNA element at its 5′ terminus. Therefore, this study was conducted for unveiling the impacts of genetically modified 5′-end motifs on SVA recovery. As a result, SVA was demonstrated to be able to repair a nucleotide-deficient 5′ terminus of its genome. The self-repairing mechanism, however, remains unclear. In addition, two online servers were jointly used to predict a high-order hairpin-pseudoknot-hairpin (HPH) structure at the 5′ terminus. This putative HPH structure was proven to be a possible key *cre* for SVA replication.

## Materials and methods

### Cell and plasmid

BSR-T7/5 cells (Buchholz et al., 1999) were cultured at 37°C with 5% CO_2_ in Dulbecco's modified Eagle's medium (DMEM), supplemented with 10% fetal bovine serum (VivaCell, Shanghai, China), penicillin (100 U/ml), streptomycin (100 μg/ml), amphotericin B (0.25 μg/ml), and G418 (500 μg/ml). The number-0 (N0) plasmid, containing an SVA cDNA clone, was genetically derived from another one that was constructed previously for rescuing a recombinant SVA (Genbank No.: KX751945.1) encoding enhanced green fluorescent protein (eGFP) *in vitro* (Liu et al., 2020). The full-length cDNA clone was regulated by the T7 promoter at its 5′ end, and contained a 30-nt-long poly(A) tail, but no ribozyme sequence at its 3′ end.

### Prediction of high-order structure at SVA 5′ terminus

The UNAFold web server (http://www.unafold.org/) is currently an amalgamation of two existing web servers: mfold and DINAMelt. The 5′-end sequence of SVA was analyzed using the UNAFold web server for modeling its RNA secondary structure. Additionally, the 5′-end sequence was subjected to pseudoknot prediction using the DotKnot method (https://dotknot.csse.uwa.edu.au/) (Sperschneider and Datta, 2010).

### Construction of 36 SVA cDNA clones

The N0 plasmid was genetically tailored to modify the 5′ terminus of the SVA cDNA clone for reconstructing 36 mutants, separately named N1 to N36, including nucleotide-mutated, -deleting, and -inserted genotypes. Overlap extension PCR (OE-PCR) and In-Fusion^®^ assembly were jointly used to construct these 36 plasmids. The first step of OE-PCR involved two rounds of PCR to amplify separately fragment I and II from the N0 plasmid using the forward primer 1 (FP1)/reverse primer 1 (RP1) and FP2/RP2, respectively (Table 1). The reaction contained 2 × PrimeSTAR Max Premix (Takara, Dalian, China), and underwent 30 cycles at 98°C (10 s), 58°C (5 s), and 72°C (3 s). Fragment I and II were separately extracted from an agarose gel after electrophoresis, and then, simultaneously used as templates for the second step of OE-PCR using the FP1/RP2. The reaction underwent 35 cycles at 98°C (10 s), 58°C (5 s), and 72°C (5 s). Fragment I and II were fused into fragment III, followed by directional cloning into the *Nde* I/*Pml* I-digested N0 plasmid for independently constructing 36 mutants using the In-Fusion^®^ Kit (Takara, Dalian, China) according to the manufacturer's instruction.

**Table 1:** Primers used to construct 5′-end-modifying SVA cDNA clones by OE-PCR.

**cDNA clone**	**FP1**	**RP1 (5^′^-3^′^)**	**FP2 (5^′^-3^′^)**	**RP2**	**PCR template**
N1	#	cccccctttcaaccctatagtgagt	actcactatagggttgaaagggggg	※	N0
N2	#	ccccccctttcaccctatagtgagt	actcactatagggtgaaaggggggg	※	N0
N3	#	gccccccctttcccctatagtgagt	actcactataggggaaagggggggc	※	N0
N4	#	ccccctttccctatagtgagtcgtattaatt	ctatagggaaagggggggctgggccctcatg	※	N0
N5	#	ccccccttccctatagtgagtcgtattaatt	ctatagggaagggggggctgggccctcatgc	※	N0
N6	#	gccccccctccctatagtgagtcgtattaat	actatagggagggggggctgggccctcatgc	※	N0
N7	#	agcccccccccctatagtgagtcgtattaat	actataggggggggggctgggccctcatgcc	※	N0
N8	#	cagccccccccctatagtgagtcgtattaat	actatagggggggggctgggccctcatgccc	※	N0
N9	#	ccagcccccccctatagtgagtcgtattaat	actataggggggggctgggccctcatgccca	※	N0
N10	#	cccagccccccctatagtgagtcgtattaat	actatagggggggctgggccctcatgcccag	※	N0
N11	#	gcatgagggcccagccccccaaaccctatagtgagtcgta	tacgactcactatagggtttggggggctgggccctcatgc	※	N0
N12	#	cccagcccaaaccctatagtgagtcgtatta	tagggtttgggctgggccctcatgcccagtc	※	N0
N13	#	gggcccagaaaccctatagtgagtcgtatta	tagggtttctgggccctcatgcccagtcctt	※	N0
N14	#	tgagggccaaaccctatagtgagtcgtatta	tagggtttggccctcatgcccagtccttcct	※	N0
N15	#	gcatgaggaaaccctatagtgagtcgtatta	tagggtttcctcatgcccagtccttcctttc	※	N0
N16	#	tgagggcccagccccccgaaagaaaccctatagtgagtc	gactcactatagggtttctttcggggggctgggccctca	※	N0
N17	#	agcccggggaaagaaaccctatagtgagtcgtatt	ggtttctttccccgggctgggccctcatgcccagt	※	N0
N18	#	caggggggggaaagaaaccctatagtgagtcgtatta	tttctttcccccccctgggccctcatgcccagtcctt	※	N0
N19	#	ccgtcgggggggaaagaaaccctatagtgagtcgtatt	ttctttcccccccgacggccctcatgcccagtccttcc	※	N0
N20	#	cgggtcgggggggaaagaaaccctatagtgagtcgtatta	ctttcccccccgacccgcctcatgcccagtccttcctttc	※	N0
N21	#	ttaccccccggaaggggaaggactgggcatga	tcatgcccagtccttccccttccggggggtaa	※	N11
N22	#	ccggaagggggactgggcatgagggcccagcc	gcccagtcccccttccggggggtaaaccggct	※	N12
N23	#	ccggaagggctgggcatgagggcccagaaacc	catgcccagcccttccggggggtaaaccggct	※	N13
N24	#	ccggaagggggcatgagggccaaaccctatag	cctcatgcccccttccggggggtaaaccggct	※	N14
N25	#	ccggaagggatgaggaaaccctatagtgagtc	tttcctcatcccttccggggggtaaaccggct	※	N15
N26	#	aaggactgggcatggtagcccagccccccct	agggggggctgggctaccatgcccagtcctt	※	N0
N27	#	aggaaggactgggcggaagggcccagccccc	gggggctgggcccttccgcccagtccttcct	※	N0
N28	#	ctgggcggagtagcccagccccccctttcaaaccc	ctgggctactccgcccagtccttcctttccccttc	※	N0
N29	#	tgggcatggcaagggcccagccccccctttcaaa	tgggcccttgccatgcccagtccttcctttcccc	※	N0
*	#	gtcttatgatagcgaaaaccctatagtgagtcgtatta	gtcttatgatagcgacccttccggggggtaaaccggct	※	N0
N30	#	agactaatgaggtagtcttatgatagcgaaaacccta	agactacctcattagtcttatgatagcgacccttccg	※	**
N31	#	cacagccggtttatttaccggaaggggaaa	tttccccttccggtaaataaaccggctgtg	※	N0
N32	#	gccggtttaccccggttaaggggaaaggaa	ttcctttccccttaaccggggtaaaccggc	※	N0
N33	#	gtttatttaggttaaggggaaaggaaggactgggcat	cccttaacctaaataaaccggctgtgtttgctagagg	※	N0
N34	#	ggatgttgctcctgacagcctctagcaaac	gtttgctagaggctgtcaggagcaacatcc	※	N0
N35	#	ctcctgacacaaactagcaaacacagccggtttaccc	gctagtttgtgtcaggagcaacatccaacctgctctt	※	N0
*	#	gtcagtctccggtttaccccccggaagggga	gtcggtttaggagcaacatccaacctgctct	※	N0
N36	#	aaccgacctagcgtcagtctccggtttaccccccg	gactgacgctaggtcggtttaggagcaacatccaa	※	**

^#^*5′-atcaagtgtatcatatgccaagtac-3′; ^※^5′-ggtggtagcaatcacgtggactctg-3′*.

*Two italic 15-bp sequences, “atcaagtgtatcata” and “ggtggtagcaatcac”, are designed for In-Fusion^®^ assembly at Nde I and Pml I sites in N0 plasmid, respectively*.

^*^The first round of PCR for amplifying two products, ^**^which are independently used as templates for the second round of PCR to construct N30 or N36 plasmid.

### Rescue of replication-competent SVAs

BSR-T7/5 cells were seeded into 24-well plates, and subsequently cultured at 37°C. Cell monolayers at 70–90% confluency were separately transfected with N0 to N36 plasmids (500 ng/well) using Lipofectamine 2000 (Thermo Fisher, Waltham, MA, United States) according to the manufacturer's instruction. The transfected cell monolayers were cultured at 37°C and observed in a randomly selected field-of-view of a fluorescence microscope at 72 h post-transfection (hpt). The cell cultures were harvested, and subjected to one round of freeze-and-thaw to collect supernatants for serial passages in the BSR-T7/5 cells. Supernatant-inoculated cell monolayers were observed in a randomly selected field-of-view of the fluorescence microscope at 48 h post-infection (hpi). The green fluorescence functioned as a reporter to indicate whether a given replication-competent SVA had been successfully rescued from its cDNA clone. If some cell monolayers always had no fluorescent phenotype at passages (P)-1, −2 and −3, it would be considered failed in virus recovery. In contrast, if successfully rescued, the recombinant SVAs would be named “rSVA-reference no. of plasmid.”

### RT-PCR detection and Sanger sequencing

Putative replication-competent rSVAs were serially passaged *in vitro*. The virus-inoculated cell cultures were harvested at 72 hpi at P6 for extracting total RNAs. The extracted products were used as templates for one-step RT-PCR analysis using the PrimeScript™ High Fidelity One-Step RT-PCR Kit (Takara, Dalian, China). The RT-PCR reaction underwent 45°C for 10 min, 94°C for 2 min, and then, 30 cycles at 98°C (10 s), 55°C (15 s), and 68°C (10 s) using FP3/RP3 or FP4/RP4 (Table 2). The RNA samples were analyzed by PCR using the same primers to identify potential plasmid residues at P6. The PCR system contained 2 × Phanta^®^ Flash Master Mix (Vazyme, Nanjing, China), and underwent 30 cycles at 98°C (10 s), 55°C (5 s), and 72°C (5 s). The RT-PCR and PCR products were analyzed by agarose gel electrophoresis. The FP4/RP4-amplified products were subjected to Sanger sequencing using the RP4.

**Table 2:** Two pairs of primers for RT-PCR analysis of rSVAs.

**Primers**	**Sequences (5^′^-3^′^)**	**Length of RT-PCR product**	rSVAs detected*
FP3	AGGCACAGAGGAGCAACATCCAA	693 bp (nt 78 to 770)	rSVA-N0 to -N25, and -N30
RP3	ATCGTTCACCGATCTAGGGTATT		
FP4	TTTGAAAGGGGGGGCTGGGC	303 bp (nt 1 to 303)	rSVA-N26 to -N29, and -N31 to -N36
RP4	CTTGCGGTTATCGCACATCT		

^*^If some rSVAs fail to be rescued from their own cDNA clones, these “viruses” would not be detected by RT-PCR.

### 5′-rapid amplification of cDNA ends (5′-RACE)

The replication-competent rSVAs were independently harvested at P9 for extracting viral RNAs, followed by RT-PCR detection as described in the preceding section. Subsequently, the RNA samples were separately subjected to a 5′-RACE reaction using the HiScript-TS 5′/3′ RACE Kit (Vazyme, Nanjing, China) according to the manufacturer's instructions. Briefly, the first strand of cDNA was synthesized from total RNA using an SVA-specific primer (SVA-P1: 5′-ATCGTTCACCGATCTAGGGTATT-3′), 5′ TS Oligo and Enzyme Mix. The first strand of cDNA was used as a PCR template for the 5′-RACE reaction. The forward and reverse primers were the Universal Primer Mix and another SVA-specific primer (SVA-P2: 5′-CGTGCGAGGGCTAAGTCTTGTAGT-3′), respectively. The 5′-RACE product was used as a template for nested PCR using the forward primer (5′-CTAATACGACTCACTATAGGGC-3′) and the SVA-P2, followed by agarose gel electrophoresis. The PCR products were extracted from an agarose gel, and then, independently subcloned into linear plasmids using the TA/Blunt-Zero Cloning Kit (Vazyme, Nanjing, China) for bacterial transformation. Single colonies were picked from each agar plate for Sanger sequencing.

### Growth kinetics of rSVAs

The replication-competent rSVAs, if proven to be genetically stable, would be measured for determining their growth kinetics *in vitro*. Briefly, the BSR-T7/5 cells were seeded into four 24-well plates (5 × 10^5^ cells/well) for incubation at 37°C for 2 h. The P4 rSVAs were separately inoculated (3 wells/progeny, 5 progenies/plate, and MOI = 0.001) into cell monolayers for incubation at 37°C. A plate was removed from the incubator at 0, 24, 48, and 72 hpi, and then subjected to one freeze-and-thaw cycle to harvest the supernatant for viral titration by TCID_50_ analysis. Briefly, the BSR-T7/5 cells were seeded into a 96-well plate, which was then cultured at 37°C for 3 h. The virus stock was serially diluted 10-fold with DMEM. The diluted virus stocks were mildly added to the 96-well plate (100 μl/well, and 8 wells/dilution), subsequently cultured at 37°C. The 96-well plate was observed using the fluorescence microscope at 48 hpi. Fluorescence-emitted wells indicated SVA-infected cell monolayers. The viral titer was estimated using the Spearman–Kärber equation (Finney, 1952). Kinetic curves of virus growth were drawn using the GraphPad Prism software (Version 8.0). Data at each time point were representative of three independent experiments.

## Results

### SVA 5′ terminus harbors a putative HPH structure

**Figure 1:**
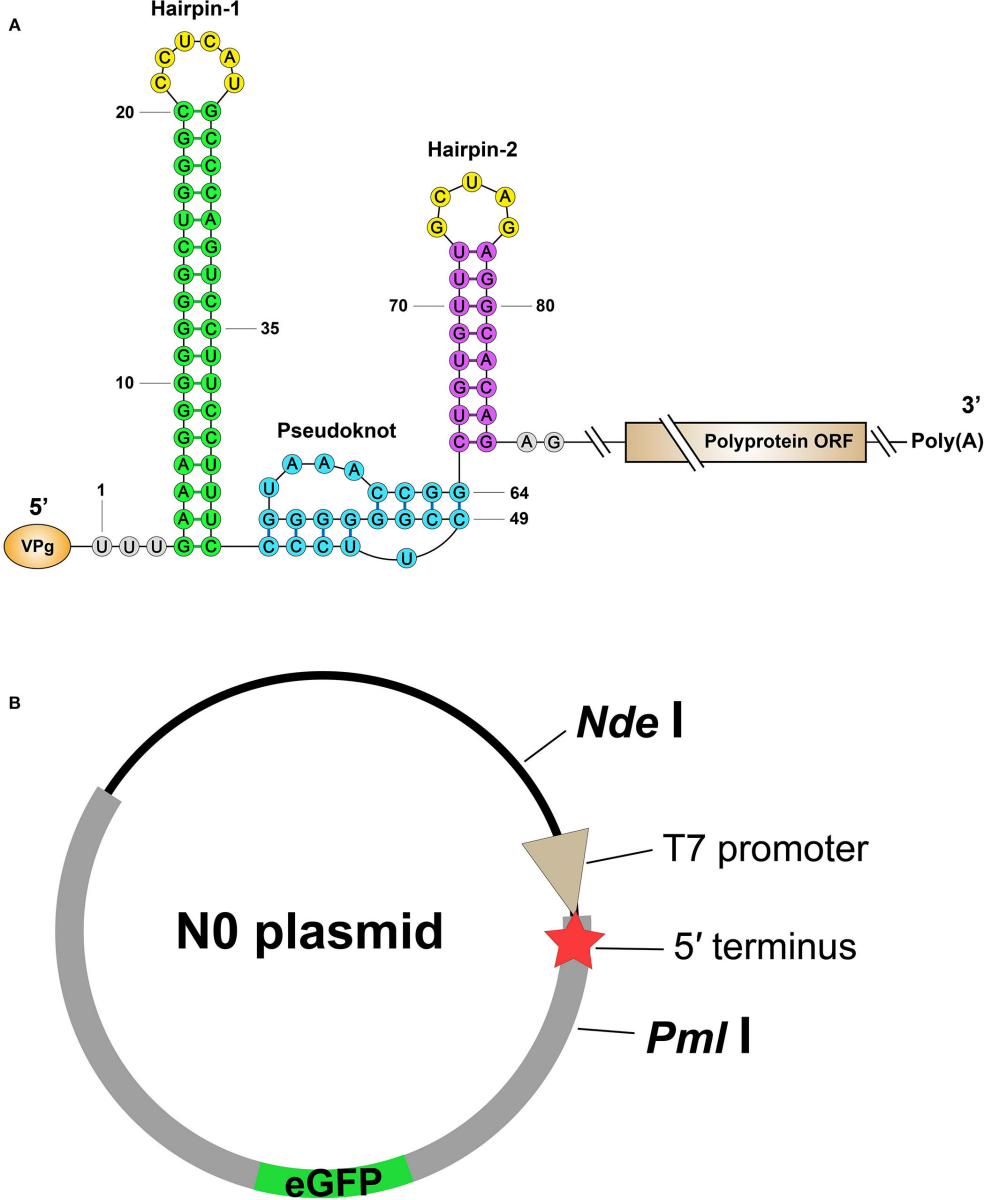
Schematic representations of putative HPH structure and wild-type cDNA clone. The HPH formation **(A)** is predicted through two online servers. The first hairpin, hairpin-1, is made up of one 17-nt-paired stem and one 6-nt-unpaired loop (Green stem-Yellow loop); the middle structure is an H-type pseudoknot, containing two stems and two loops (Blue stem-Blue loop); the second hairpin, hairpin-2 (Purple stem-Yellow loop), is structurally simpler than the hairpin-1. The N0 plasmid contains the wild-type SVA cDNA clone with a fusion sequence of eGFP-*Thosea asigna* virus 2A **(B)**. The red star indicates the 5′ terminus of cDNA clone.

Figure 1A schematically shows the SVA genome that was composed of 5′ UTR, polyprotein ORF, 3′ UTR, and poly(A) tail. The first three nucleotides were “UUU” at the 5′ end (Figure 1A, gray circle-marked). The 5′-end motif between nt 4 and 85 was computationally predicted to form an HPH structure: the first hairpin (hairpin-1) was made up of one 17-nt-paired stem and one 6-nt-unpaired loop (Figure 1A, green stem-yellow loop); the middle structure was an H-type pseudoknot, containing two stems and two loops (Figure 1A, blue stem-blue loop); the second hairpin (hairpin-2) (Figure 1A, purple stem-yellow loop) was structurally simpler than the hairpin-1.

### A total of thirty-six mutated cDNA clones are constructed

**Figure 2:**
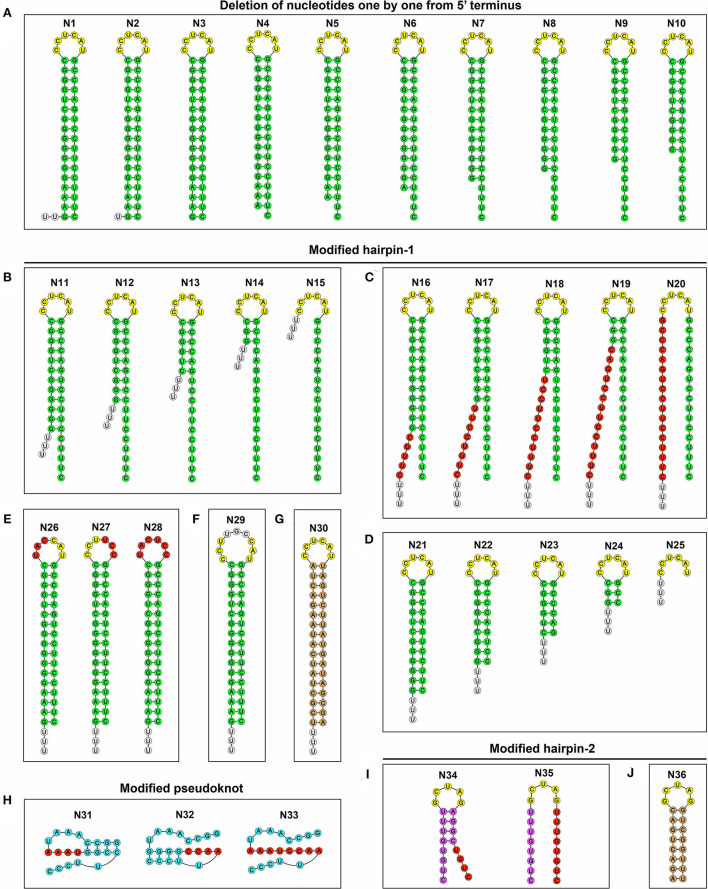
Schematic representations of 36 putative RNA structures, corresponding to N1 to N36 genotypes. The first ten nucleotides, “TTTGAAAGGG,” are deleted one by one from the N0 to construct N1 to N10 **(A)**. The HPH-forming motif (nt 4 to 85) in the N0 is genetically modified to construct N11 to N36, including single-stranded deletion **(B)**, single-stranded mutation [**(C,E,H,I)**, Red circle-marked], double-stranded deletion **(D)**, double-stranded substitution [**(G,J)**, Brown circle-marked] and single-stranded insertion [**(F)**, Gray circle-marked].

The eGFP-tagged SVA cDNA clone is schematically shown in Figure 1B. The N0 plasmid was genetically modified to reconstruct 36 mutants, N1 to N36, using OE-PCR and In-Fusion^®^ assembly. The 36 mutants contained different modified motifs at the 5′ terminus. The first ten nucleotides were deleted one by one to construct N1 to N10, corresponding to 1- to 10-nt-deleting genotypes (Figure 2A). The HPH-forming motif between nt 4 and 85 was genetically modified for constructing N11 to N36, each of which would partially or totally disrupt hairpin-1, pseudoknot, or hairpin-2 formation. The N11 to N36 included single-stranded deletion (Figure 2B), single-stranded mutation (Figures 2C,E,H,I, red circle-marked), double-stranded deletion (Figure 2D), double-stranded substitution (Figures 2G,J, brown circle-marked) and single-stranded insertion (Figure 2F, gray circle-marked). All 36 plasmids were subjected to Sanger sequencing for confirming their mutation identities.

### Nine groups have fluorescence-emitted phenotypes with blind passaging

**Figure 3:**
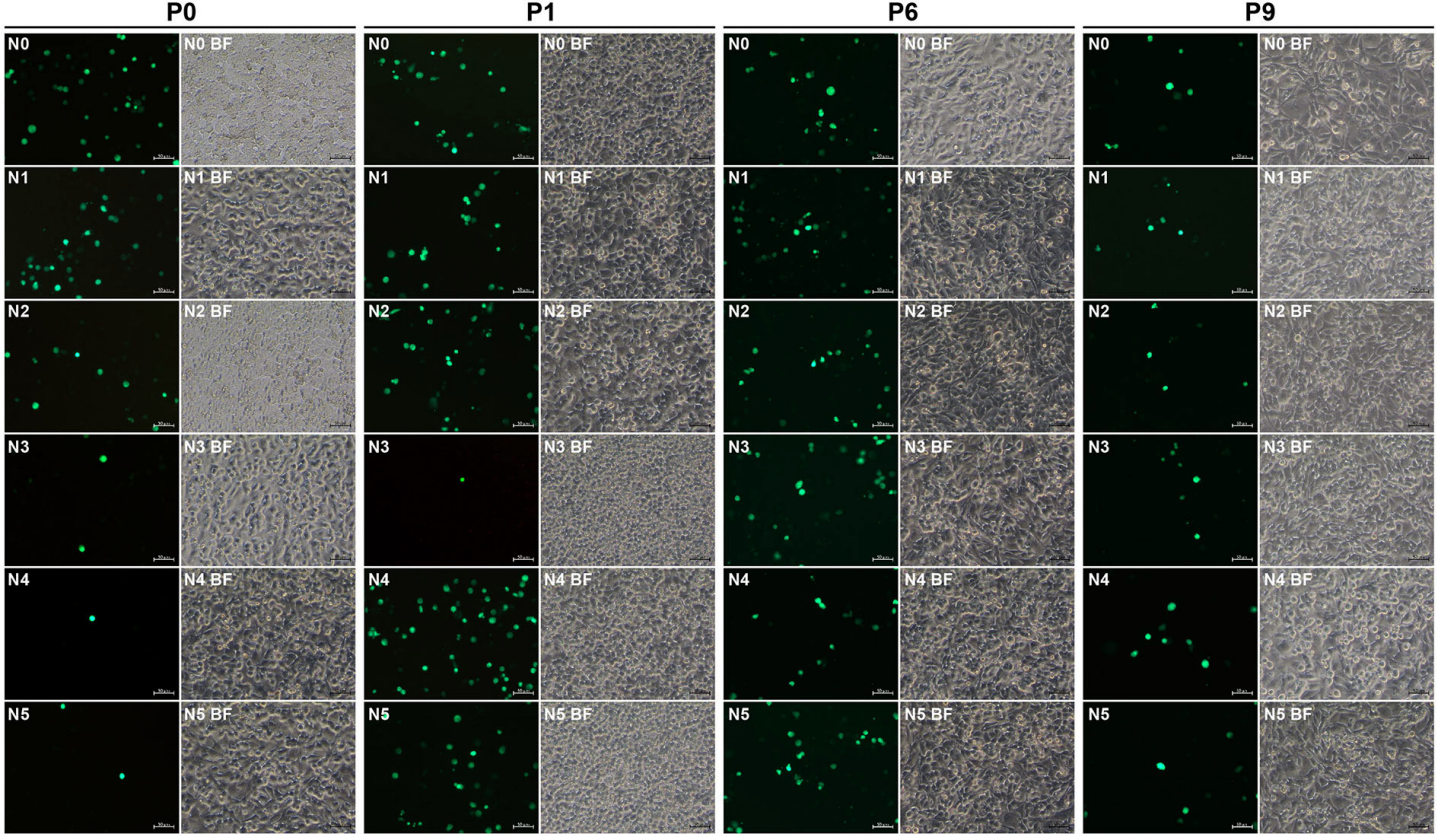
Rescue and passaging of rSVA-N0 to -N5. Green fluorescence is visible on cell monolayers during serial blind passaging. BF, bright field. P0: passage-0 at 72 hpt. P1, P6 and P9: passage-1, −6, and −9 at 48 hpi. Bar = 50 μm.

**Figure 4:**
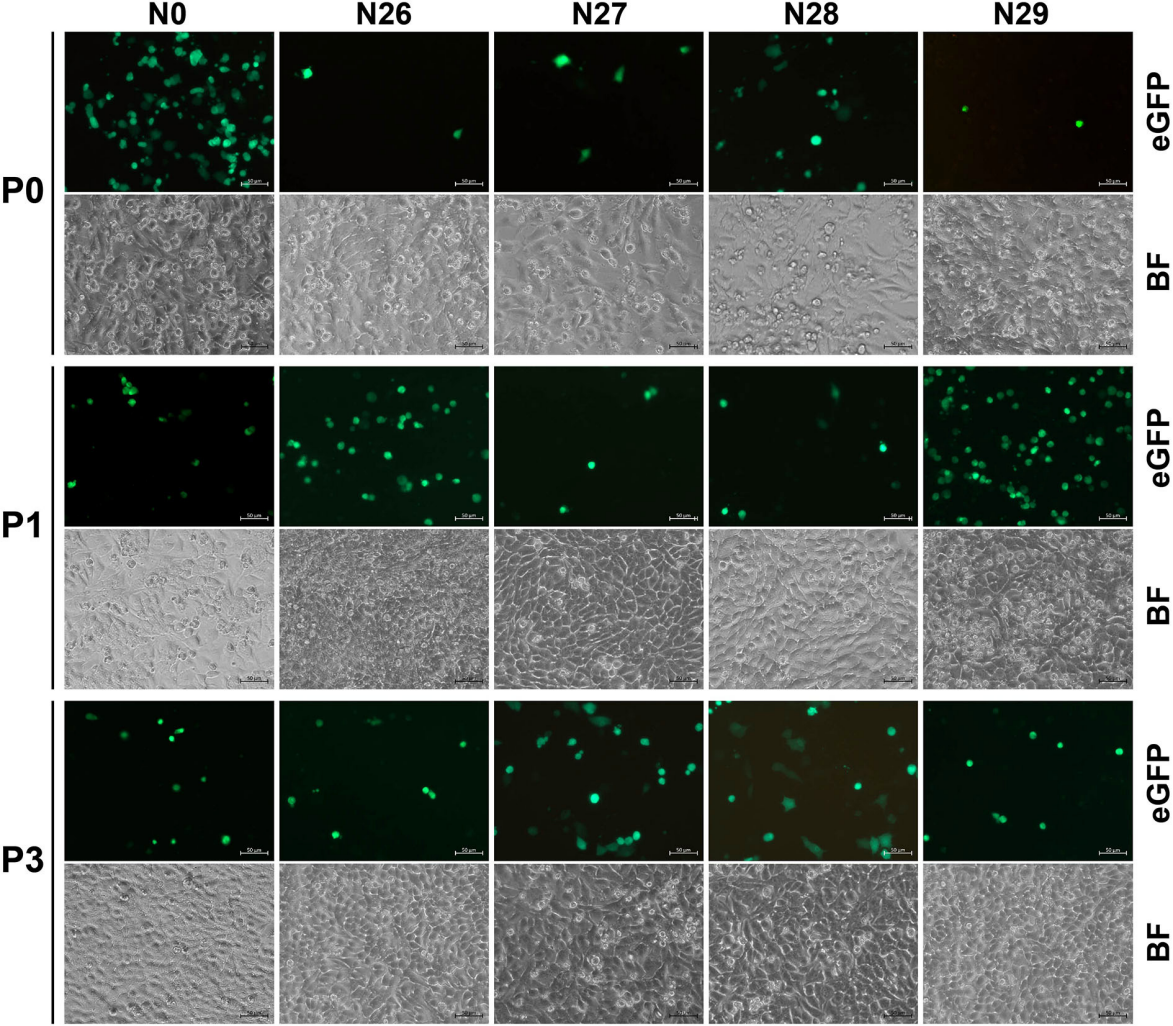
Rescue and passaging of rSVA-N26 to -N29, and -N0. Green fluorescence is visible on cell monolayers during serial blind passaging. BF, bright field. P0: passage-0 at 72 hpt. P1 and P3: passage-1 and −3 at 48 hpi. Bar = 50 μm.

The N0 to N36 were independently transfected into the BSR-T7/5 cells to rescue replication-competent rSVAs. Green fluorescence was observable on all plasmid-transfected cell monolayers at 72 hpt (Figures 3, 4, Panel P0; Supplementary Figures 1–4, Panel P0), because such vital wild-type elements as the IRES sequence were not disrupted in any of the 36 cDNA clones. Nevertheless, besides the group N0, only the groups N1, N2, N3, N4, N5, N26, N27, N28, and N29 always showed their fluorescence-emitted phenotypes through blind passaging (Figures 3, 4; Supplementary Figures 1–4). The green fluorescence, functioning as a reporter of virus recovery, preliminarily demonstrated that rSVA-N1 to -N5 and -N26 to -N29 were rescued from their cDNA clones.

### RT-PCR confirms that only nine rSVAs are rescued

**Figure 5:**
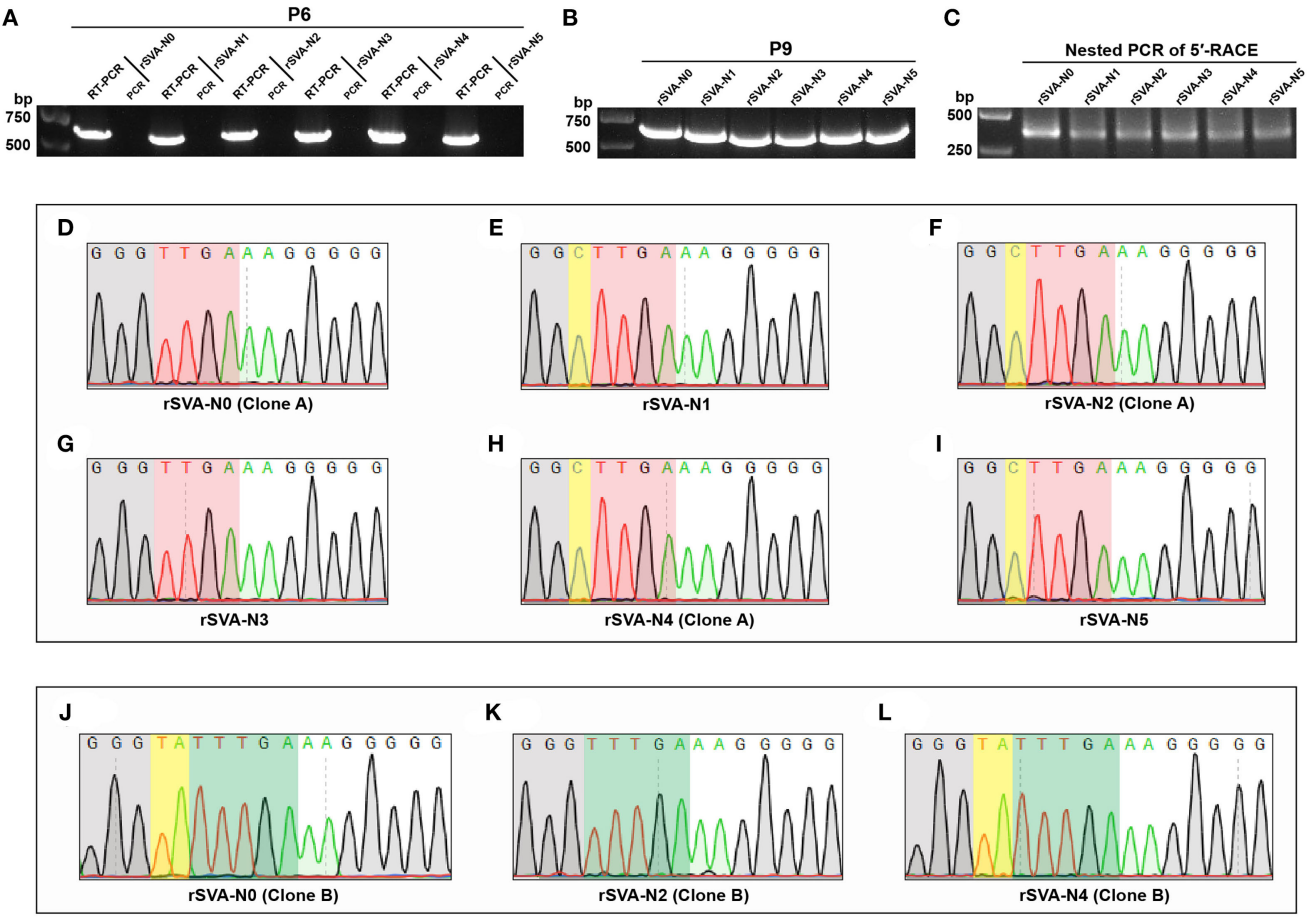
RT-PCR detection, 5′-RACE analysis and Sanger sequencing. RT-PCR detection of rSVA-N0 to -N5 at P6 **(A)**. PCR controls are assigned to demonstrate no interference of cDNA residues. RT-PCR detection of rSVA-N0 to -N5 at P9 **(B)**. Nested PCR amplification for 5′-RACE products of rSVA-N0 to -N5 at P9 **(C)**. Sanger sequencing chromatograms of 5′-end sequences of rSVA-N0 to -N5 at P9 **(D–L)**. The “TTGA” and “TTTGA” genotypes are covered with red and green shadows, respectively. The partial 3′-end sequences of adapters are covered with gray shadows. The yellow shadows indicate one or two extra nucleotides inserted between the 3′ end of adapter and the 5′ end of viral cDNA.

**Figure 6:**
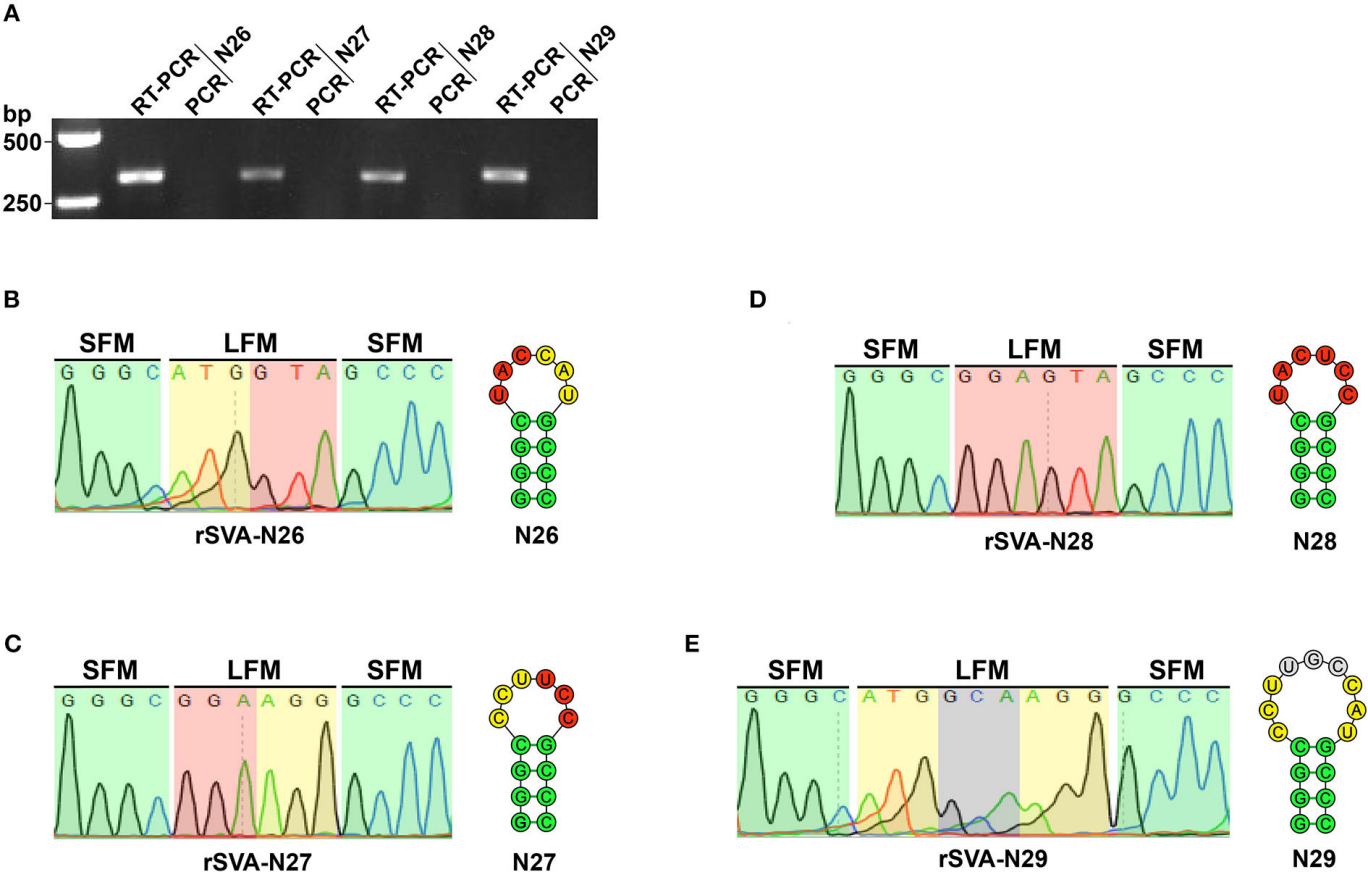
Detection and characterization of rSVA-N26 to -N29. RT-PCR analysis of rSVA-N26 to -N29 at P6 **(A)**. PCR controls are assigned to demonstrate no interference of plasmid residues. Sanger sequencing for hairpin-1-forming motifs of rSVA-N26 to -N29 at P6 **(B–E)**. Since the reverse primer (RP4) is used for Sanger sequencing, four chromatogram-exhibited fragments are reverse-complement sequences of hairpin-1 motifs. Green, yellow, red and gray shadow-covered chromatograms (rSVA-N26 to -N29) match to green, yellow, red and gray circle-marked fragments in the hairpin-1 (N26 to N29), respectively. LFM, loop-forming motif; SFM, stem-forming motif.

The viral progenies of groups N0 to N5 and N26 to N29 were harvested at P6 for RT-PCR detection, showing 693 (Figure 5A)- and 303 (Figure 6A)-bp expected bands, respectively. At the same time, the PCR analysis indicated no cDNA clone contamination affecting RT-PCR detection (Figures 5A, 6A). The RT-PCR results confirmed that rSVA-N1 to -N5 and -N26 to -N29 were successfully rescued from their cDNA clones.

### rSVA-N26 to -N29 have genetically stable loop-forming motifs

The RT-PCR products of rSVA-N26 to -N29 underwent Sanger sequencing using the reverse primer (Table 2, RP4). The result showed that all four P6 progenies retained their original genotypes (Figures 6B–E), *i.e*., genetically modified loop motifs in the N26 to N29 plasmids (Figures 2E,F), implying the wild-type loop motif (CCUCAU) was not a high-fidelity element in hairpin-1 for SVA replication.

### SVA is able to repair a nucleotide-deficient 5′ terminus

The P9 rSVA-N0 to -N5 were also subjected to RT-PCR analysis, still showing expected bands on the gel (Figure 5B). A total of six samples of total RNA (rSVA-N0 to -N5) were separately subjected to a 5′-RACE reaction at P9. The result of the nested PCR showed approximately 400-bp-long bands on the agarose gel (Figure 5C). A total of six products of nested PCR were purified for constructing recombinant plasmids, followed by bacterial transformation. A total of six single colonies were picked for Sanger sequencing. Figures 5D–L show representative sequencing chromatograms of 5′-terminus sequences within viral cDNAs. Untypical chromatograms are not shown here. The result of Sanger sequencing indicated that each of the recombinants had a wild-type (Figures 5J–L, green-shadowed) or (and) wild-type-like (Figures 5D–I, red-shadowed) 5′-terminus sequence. Irrespective of the number of single nucleotides deleted from the N0 plasmid, all five nucleotide-deleting rSVAs could repair nucleotide defects in their individual 5′ termini.

### rSVA-N26 to -N29 have similar growth kinetics to that of rSVA-N0

**Figure 7:**
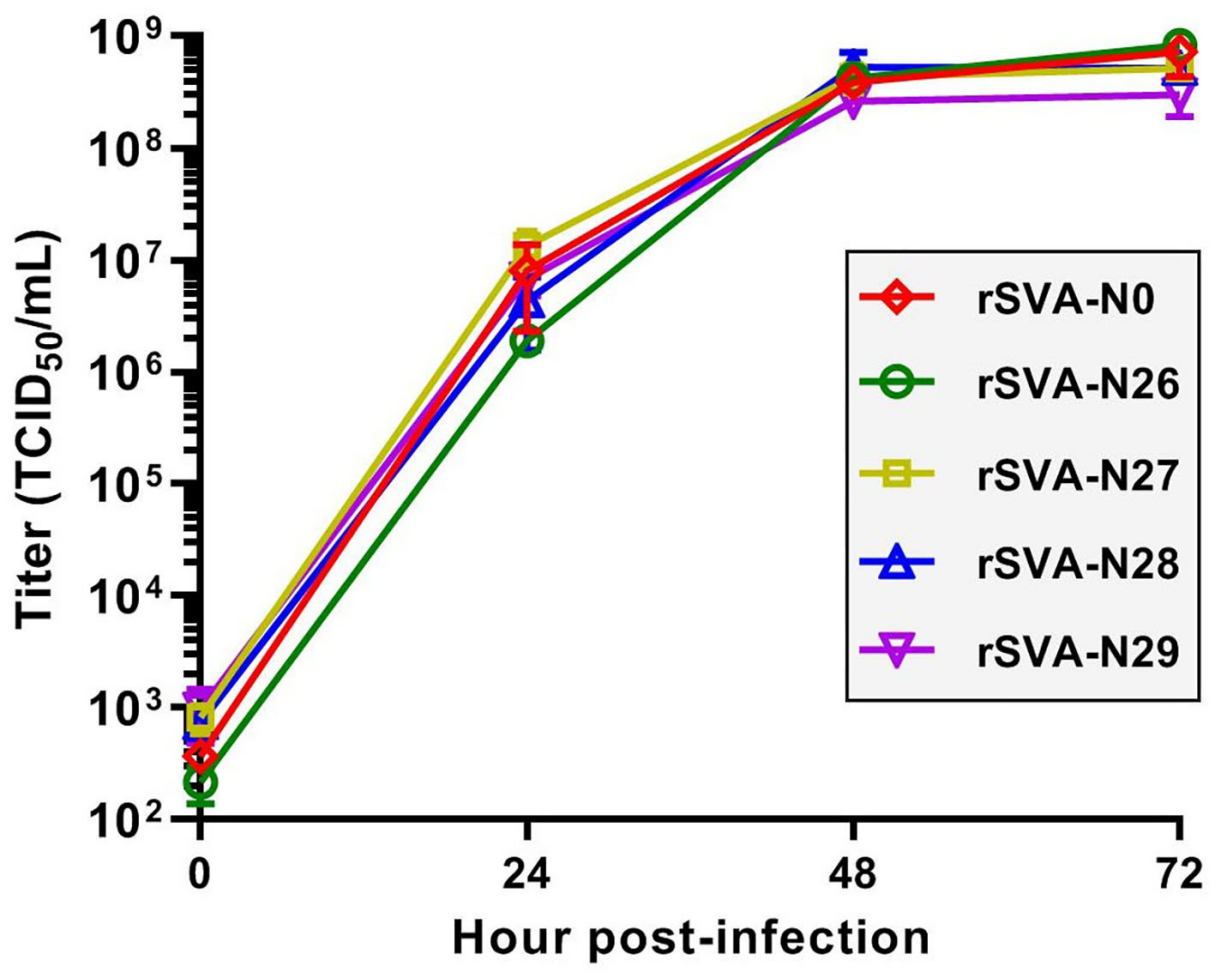
Multistep growth curves of rSVAs at P4. Data at 0, 24, 48 and 72 hpi are representative of three independent experiments. Error bar indicates standard deviation.

A total of nine rSVAs were rescued successfully, whereas only the rSVA-N26 to -N29 were genetically stable in their mutated motifs. To compare their growth kinetics with that of the rSVA-N0 *in vitro*, the BSR-T7/5 cell monolayers were inoculated with the viral progenies at MOI of 0.001. Viral titers were measured at 0, 24, 48, and 72 hpi for drawing their growth curves at P4 (Figure 7). The result showed that rSVA-N26 to -N29 had similar growth kinetics to that of rSVA-N0. All peak titers appeared at 72 hpi, more than 10^8^ TCID_50_/ml. Among all five progenies, rSVA-N26 induced the lowest and highest titers at 0 and 72 hpi, respectively.

## Discussion

*Picornaviridae* is a well-characterized family within the plus-strand RNA viruses. Picornaviruses can infect a variety of vertebrates, including fish, mammals, and birds. Their genomes generally range from 7,500 to 8,000 nt in length. The VPg is covalently linked to the picornaviral 5′ end. SVA is an emerging picornavirus. Its VPg is predicted to be a 22-aa-long protein (Liu et al., 2021a). Since VPg-pUpU functions as a protein primer to initiate replication of the picornaviral genome, the first two nucleotides must be “UU” at the extreme 5′ end of SVA. Poliovirus is a model picornavirus, requiring a precise 5′ terminus for efficient synthesis of plus-strand RNA. Non-self-nucleotides ahead of its 5′ terminus would be trimmed during poliovirus replication (van der Werf et al., 1986; Herold and Andino, 2000). Our preliminary experiment also demonstrated that a few extra nucleotides, if added to the 5′ terminus of the SVA cDNA clone, would be removed from the genome of rescued SVA (data not shown). Interestingly, a previous report showed that deletion of two single nucleotides from the extreme 5′ end of hepatitis A virus (HAV, picornavirus) abolished the infectivity of HAV RNA, whereas the poliovirus RNA lacking the first two 5′-terminus residues was still infectious (Harmon et al., 1991).

We had established previously a reverse genetics system of eGFP-tagged SVA (Liu et al., 2020), useful for demonstrating whether a given motif was required for virus propagation (Liu et al., 2021b). This study was divided into two major parts. The first part aimed to explore whether the SVA was able to repair nucleotide defects in its 5′ terminus. The first ten nucleotides were “TTTGAAAGGG” at the extreme 5′ end of the wild-type cDNA clone. We speculated that the deletion of the first two (TT) or more nucleotides would severely affect or even abolish virus rescue. In contrast to our speculation, rSVA-N1 to -N5 were recovered successfully from their cDNA clones.

Some rescued viruses, like the rSVA-N3 (Figure 3), induced dim fluorescence on cell monolayers at P0 or P1. A low proportion of fluorescent cells was possibly attributed to unnormalized virus titers, the choice of the cell line, low transfection efficiency, or (and) insufficient infection time. Beyond that, a more important factor might be the nucleotide-deficient 5′ end that exerted an impact on the replication of rescued viruses at P0 or P1. Such an impact, nonetheless, was gradually eliminated with serial passaging, perhaps due to the nucleotide-deficient 5′ end that was slowly repaired. Conversely, if the nucleotide-deficient motifs had been genetically stable with viral passaging, a few nucleotides would be unnecessary at the extreme 5′ end for SVA propagation. The 5′-RACE analysis revealed that these so-called mutants actually reverted to the wild-type or (and) wild-type-like genotype (Figures 5D–L). Specifically, six sequencing chromatograms generally contained unspecific maps, up to one to three (data not shown), for a single 5′-RACE product. The specific chromatograms (up to three to five) consisted of either wild-type or wild-type/wild-type-like genotypes.

The RACE kit contained an adapter, 5′ TS Oligo, used for the synthesis of first strand cDNA. The 3′ end of adapter contained a few consecutive “G” residues and was covalently linked to the 5′ terminus of viral cDNA. The partial 3′-end sequences of the adapters were covered with gray shadows in Figures 5D–L. Although “GGG” was added to the 3′ end of the T7 promoter within the full-length cDNA clone to enhance transcription efficiency (Martin et al., 1988), the gray-shadowed “G” residues in Figures 5D–L were derived from the adapter, rather than from cDNA clones. Interestingly, we found that one or two extra nucleotides (Figures 5E,F,H–J,L, yellow-shadowed) were inserted between the 3′ end of adapter and the 5′ end of viral cDNA. In theory, such unexpected insertions did not belong to the 5′-terminus motif of viral cDNA, because the 5′ terminus of picornavirus was a highly conserved structure (VPg-pUpU) (Sun et al., 2014). Alternatively, the 1- or 2-nt-inserted structure might be attributed to problematic adapter ligation.

Three major RNA elements play a crucial role in the initiation of picornaviral RNA replication. They are separately one high-order structure at the 5′ end, one poly(A) tail at the 3′ end, and one *cre* located at a given site that depends on different picornaviruses (Paul and Wimmer, 2015). A short motif at the 5′ terminus of the picornaviral genome generally self-forms into a high-order RNA structure that may be an important *cre* for RNA replication (Brown et al., 1991; Le et al., 1993; Barton et al., 2001; Nagashima et al., 2005). Regarding these structures, enteroviruses were the best-characterized members in the family *Picornaviridae*. For instance, a cloverleaf structure at the 5′ terminus of the polioviral genome can bind viral and cellular proteins, closely involved in RNA replication (Barton et al., 2001). Other picornaviruses have been also demonstrated to bear essential 5′-end structures (for a review, see Liu et al., 2009).

Unfortunately, the SVA is less well-defined with respect to its terminal structures. A high-order HPH structure, albeit computationally predicted to exist at the 5′ terminus of the SVA genome, was experimentally unconfirmed. This prompted us to conduct the second part of this study to preliminarily uncover whether such a putative RNA structure functioned as a *cre* for SVA replication. Another set of plasmids (N11 to N36) was designed and constructed in an attempt to rescue the HPH-deformed rSVAs using reverse genetics. If a given SVA is rescued successfully from its cDNA clone with a mutated motif, and more importantly, if the mutated motif is genetically stable with viral passaging, it can be concluded that the wild-type motif is not a high-fidelity element during SVA replication. Otherwise, it would be a *cre*, unable to tolerate a single or multiple point mutations.

We found that all stem regions were indispensable in the putative HPH structure for SVA recovery. Irrespective of single-stranded deletion (Figure 2B), single-stranded mutation (Figures 2C,H,I), double-stranded deletion (Figure 2D), or double-stranded substitution (Figures 2G,J), replication-competent rSVAs failed to be rescued from any of the cDNA clones with disrupted stem regions. As mentioned in the subheading Introduction, the Aichi virus also has a 5′-end HPH structure, slightly different from that of SVA. It acts as a *cre* not only for viral RNA encapsidation (Sasaki and Taniguchi, 2003) but also for positive- and negative-strand RNA synthesis (Nagashima et al., 2005). Especially, the interaction between polypeptide 3ABC and the 5′-end structure is involved in the synthesis of the Aichi virus negative-strand RNA (Nagashima et al., 2008). We speculate here that the 5′-end HPH structure of SVA may also play a role in negative-strand RNA synthesis or (and) viral encapsidation. Such speculation remains to be clarified.

The cloverleaf structure at the 5′ end of the enteroviral genome is a crucial element in genomic replication. The cloverleaf structure of human rhinovirus isotype 14 (enterovirus) harbors a stem-loop B that contains an 8-nt loop. There are four consecutive pyrimidine bases located in the loop region. Such a pyrimidine-rich motif increases the flexibility of 8-nt loop, resulting in all or some of these nucleotides that can be immediately accessible for protein interactions. The pyrimidine-rich loop has been demonstrated to be an apparent recognition site for the polyC-binding protein in the host (Warden et al., 2017). To test whether the wild-type hairpin-1 loop was required for SVA propagation or not, we constructed four SVA cDNA clones with modified hairpin-1 loops for virus recovery. Unexpectedly, the rSVA-N26 to -N29 were successfully rescued and showed their loop-forming motifs genetically stable during serial passaging (Figures 6B–E). Moreover, the four rSVAs had similar growth kinetics to that of rSVA-N0 (Figure 7). These pieces of evidence suggest the possibility that the hairpin-1 loop specifically interacts neither with viral nor with cellular proteins in cells.

In conclusion, SVA had an instinct of repairing a nucleotide-deficient 5′ terminus. The limit of self-repairing is five consecutive nucleotides, whereas the mechanism remains to be clarified. The HPH structure was computationally predicted to form at the 5′ terminus of the SVA genome. Its formation was possibly a crucial *cre* for such viral mechanisms as SVA RNA synthesis. If so, it would interact with the viral or (and) host proteins, commonly known as *trans*-acting factors, for initiating RNA replication. What are *trans*-acting factors, and how the *cis*-acting element interacts with them, remain to be elucidated.

## Data availability statement

The original contributions presented in the study are included in the article/Supplementary materials, further inquiries can be directed to the corresponding authors.

## Author contributions

FL conducted experiments and wrote the manuscript. HM and QW performed the construction of plasmids, recovery of rSVAs, and virus titration. ML, ZL, and XH carried out RT-PCR detection. DZ and YD completed a 5′-RACE analysis. SL, FZ, and JC were in charge of data analysis. BN, HS, and FL provided funding. All authors read and approved the manuscript.

## Funding

This work was supported by the Expert Fund from the Shandong Technique System of Pig Industry for Disease Control (SDAIT-08-07), the National Key R&D Program for the 14th Five-Year Plan (2021YFD1800301-04), and the Postgraduate Innovation Program of Qingdao Agricultural University (QNYCX22001).

## Conflict of interest

The authors declare that the research was conducted in the absence of any commercial or financial relationships that could be construed as a potential conflict of interest.

## Publisher's note

All claims expressed in this article are solely those of the authors and do not necessarily represent those of their affiliated organizations, or those of the publisher, the editors and the reviewers. Any product that may be evaluated in this article, or claim that may be made by its manufacturer, is not guaranteed or endorsed by the publisher.

## Supplementary material

Supplementary Figure 1 Rescue and passaging of “rSVA-N6 to -N10”. Green fluorescence is unobservable on cell monolayers at P3 and P4. BF, bright field. Bar = 50 μm. P0: passage-0 at 72 hpt. P1, P3 and P4: passage-1, −3 and −4 at 48 hpi.

Supplementary Figure 2 Rescue and passaging of “rSVA-N11 to -N25 and -N30”. Green fluorescence is unobservable on cell monolayers at P1 and P3. BF, bright field. Bar = 50 μm. P0: passage-0 at 72 hpt. P1 and P3: passage-1 and −3 at 48 hpi.

Supplementary Figure 3 Rescue and passaging of “rSVA-N31 to -N33”. Green fluorescence is unobservable on cell monolayers at P1 and P3. BF, bright field. Bar = 50 μm. P0: passage-0 at 72 hpt. P1 and P3: passage-1 and −3 at 48 hpi.

Supplementary Figure 4 Rescue and passaging of “rSVA-N34 to -N36”. Green fluorescence is unobservable on cell monolayers at P1 and P3. BF, bright field. Bar = 50 μm. P0: passage-0 at 72 hpt. P1 and P3: passage-1 and −3 at 48 hpi.
