# Supplementary figures and images for: The 5′-end motif of Senecavirus A cDNA clone is genetically modified in 36 different ways for uncovering profiles of virus recovery
Source: Front Microbiol. 2022 Aug 17;13:957849. doi: 10.3389/fmicb.2022.957849 (PMC9428520; doi:10.3389/fmicb.2022.957849)

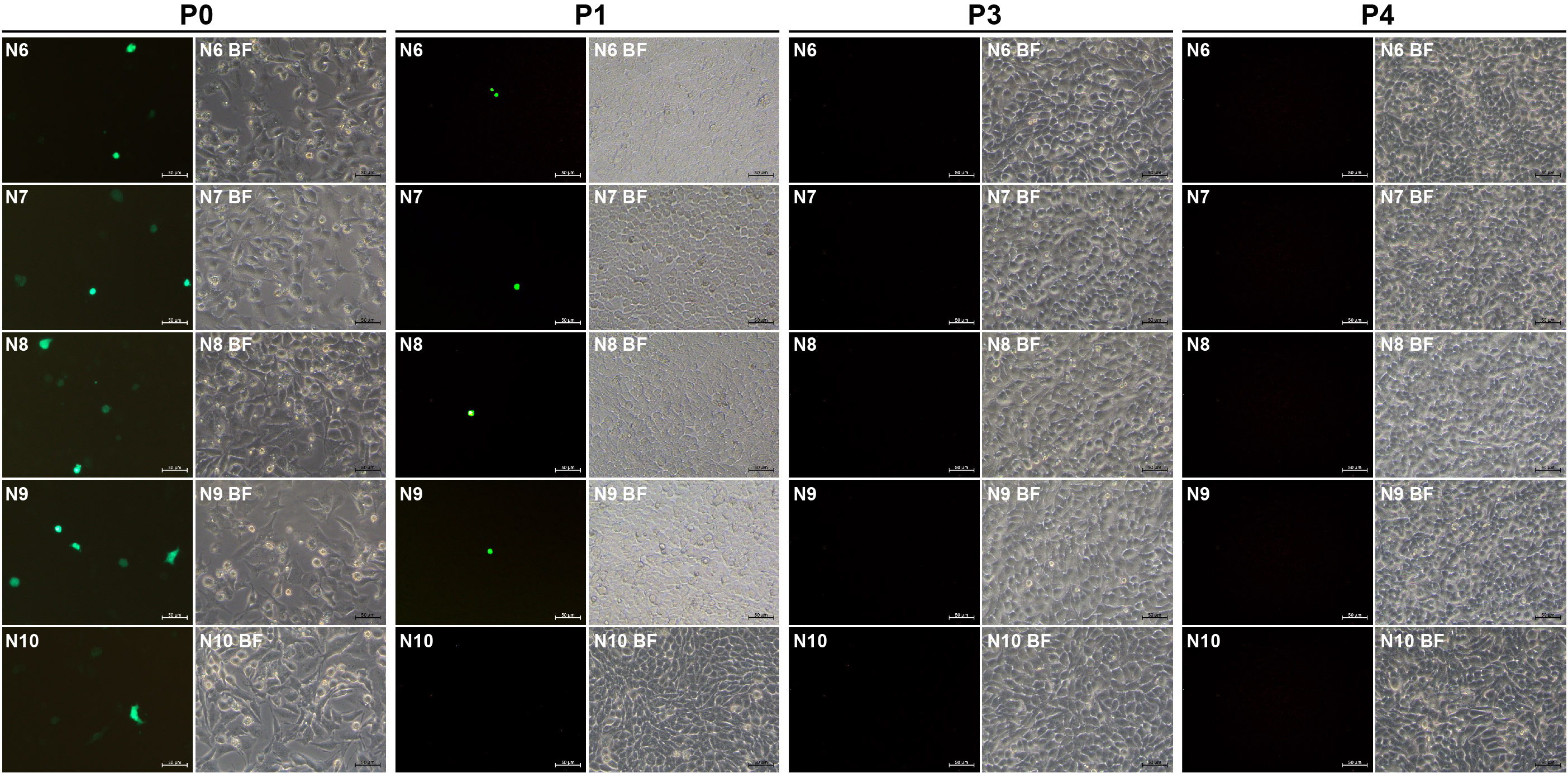

Supplement: Supplementary Figure 1 — Rescue and passaging of “rSVA-N6 to -N10”. Green fluorescence is unobservable on cell monolayers at P3 and P4. BF, bright field. Bar = 50 μm. P0: passage-0 at 72 hpt. P1, P3 and P4: passage-1, −3 and −4 at 48 hpi. [file Image_1.JPEG]

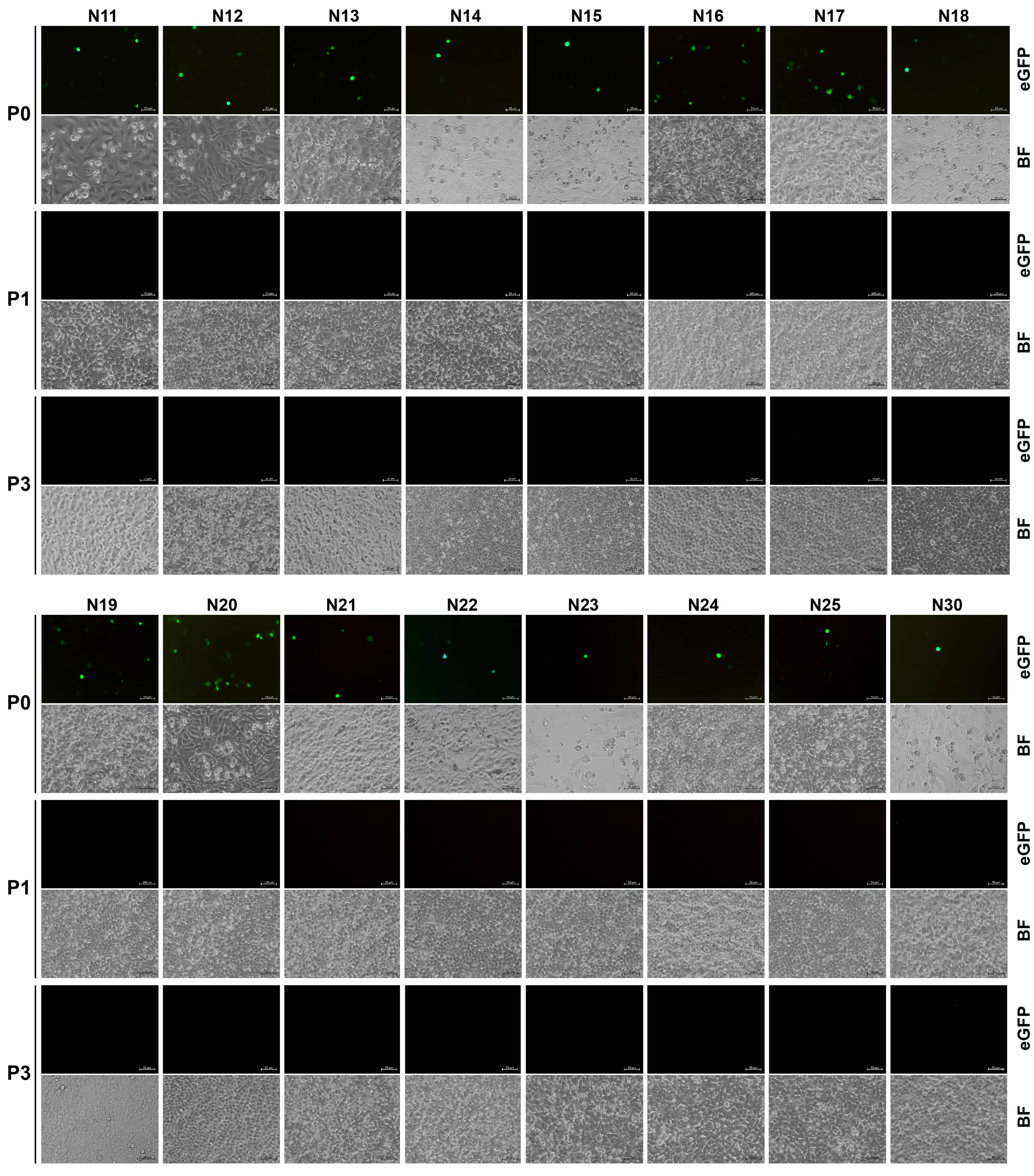

Supplement: Supplementary Figure 2 — Rescue and passaging of “rSVA-N11 to -N25 and -N30”. Green fluorescence is unobservable on cell monolayers at P1 and P3. BF, bright field. Bar = 50 μm. P0: passage-0 at 72 hpt. P1 and P3: passage-1 and −3 at 48 hpi. [file Image_2.JPEG]

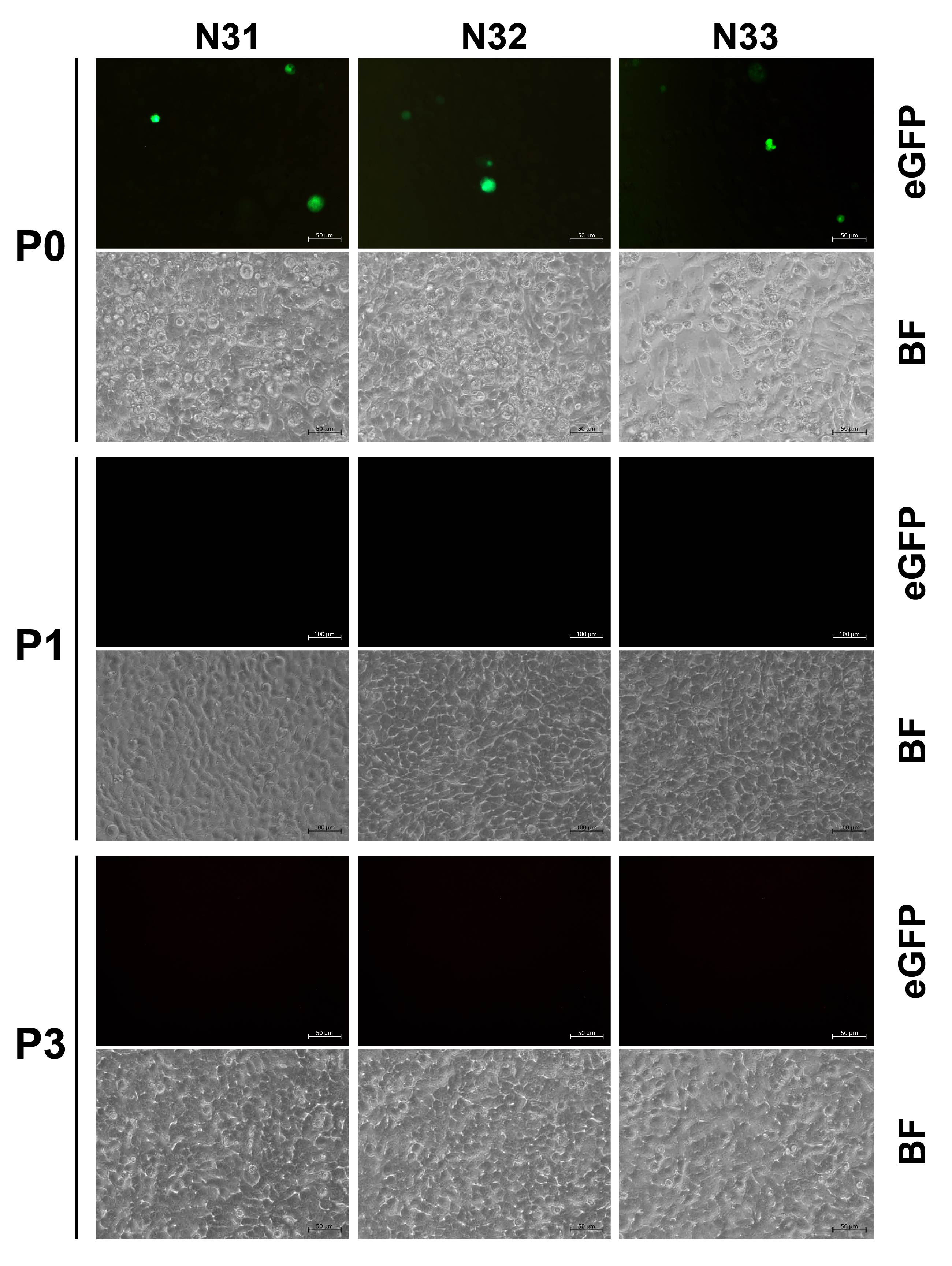

Supplement: Supplementary Figure 3 — Rescue and passaging of “rSVA-N31 to -N33”. Green fluorescence is unobservable on cell monolayers at P1 and P3. BF, bright field. Bar = 50 μm. P0: passage-0 at 72 hpt. P1 and P3: passage-1 and −3 at 48 hpi. [file Image_3.JPEG]

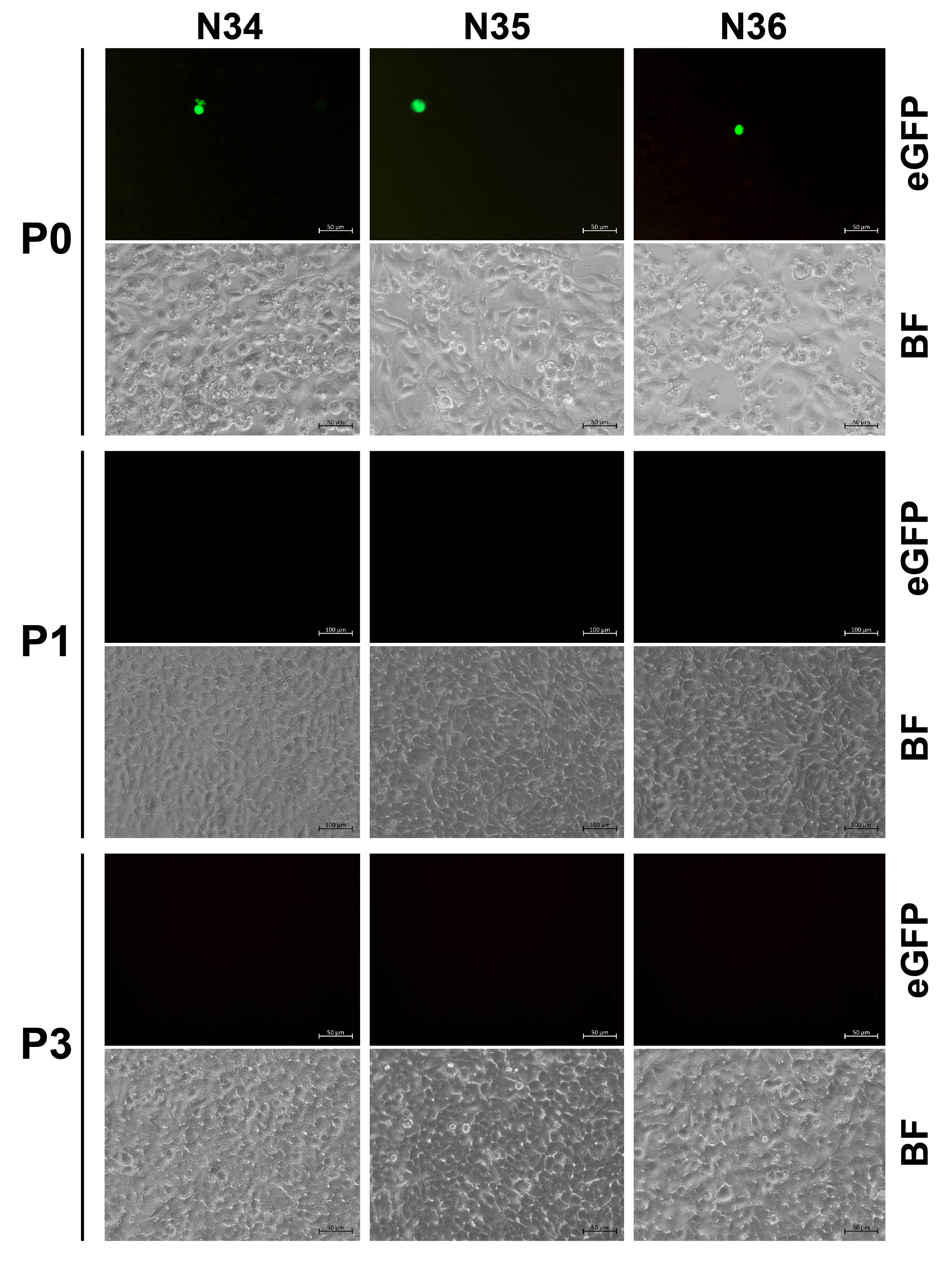

Supplement: Supplementary Figure 4 — Rescue and passaging of “rSVA-N34 to -N36”. Green fluorescence is unobservable on cell monolayers at P1 and P3. BF, bright field. Bar = 50 μm. P0: passage-0 at 72 hpt. P1 and P3: passage-1 and −3 at 48 hpi. [file Image_4.JPEG]
